# Understanding the Role of Academic Partners as Technical Assistance Providers: Results from an Exploratory Study to Address Precarious Work

**DOI:** 10.3390/ijerph16203903

**Published:** 2019-10-15

**Authors:** Tessa Bonney, Christina Welter, Elizabeth Jarpe-Ratner, Lorraine M. Conroy

**Affiliations:** Environmental and Occupational Health Sciences, School of Public Health, University of Illinois at Chicago, Chicago, IL 60607, USA

**Keywords:** precarious work, action learning, technical assistance, community-university partnership, policy, systems, and environmental (PSE) change

## Abstract

Universities may be well poised to support knowledge, skill, and capacity-building efforts to foster the development of multi-level interventions to address complex problems. Researchers at the University of Illinois at Chicago (UIC) engaged organizations interested in developing policy- and systems-level initiatives to address the drivers of precarious work in a six-meeting Action Learning (AL) process, in which the researchers served as technical assistance (TA) providers focused on facilitating learning and promoting critical thinking among participants. This exploratory qualitative study examined the role, facilitators, challenges, and impacts of university facilitation in this context. A total of 22 individuals participated in this study, including UIC TA providers, content expert TA providers from labor-focused organizations, and TA recipients from health-focused organizations. Results from interviews and a focus group highlight the utility of a university connecting organizations from different disciplines that do not traditionally work together, and suggest that the TA provided by UIC helped participants think concretely about precarious work and ways in which their organizations might work collaboratively to bring about sustainable change. Findings from this study suggest that university facilitation using an AL approach may be effective in increasing knowledge to action.

## 1. Introduction

In recent years, the National Institute for Occupational Safety and Health (NIOSH) has funded several *Total Worker Health*^®^ Centers for Excellence at universities across the United States with the goal of building scientific evidence around innovative approaches to address complex problems faced by workers in the United States [1]. Occupational safety and health researchers and practitioners are increasingly called to navigate the complexities of a changing work landscape, in which work arrangements have increasingly shifted away from standard, full-time employment with benefits toward non-standard, “atypical”, and precarious work arrangements such as employment in temporary or contract jobs [2]. The University of Illinois at Chicago (UIC) Center for Healthy Work, one of the NIOSH Total Worker Health (TWH) Centers for Excellence, has focused its efforts on understanding the barriers faced by workers in these precarious jobs in Illinois, and building evidence around the development of interventions to remove those barriers [3]. 

Over the past several years, a subset of researchers at the UIC Center for Healthy Work have engaged with individuals and organizations in Chicago and across the state of Illinois to better understand the causes and consequences of precarious work and initiatives that are already underway to address them. One of the Center for Healthy Work’s aims is to work with a variety of organizational partners, across sectors and levels, to build organizations’ capacities to develop and implement interventions to address the barriers to healthy work. While some studies have examined the value and impacts of community–university engagement in research and practice partnerships [4,5,6], existing studies have not focused on universities as a convener for processes focused heavily on planning and preparing for action, and focused less on traditional research methodology. The UIC Center for Healthy Work is examining the role that a university can play in supporting knowledge, skills, and overall capacity-building efforts to foster the development of multi-level initiatives to address precarious work. 

### 1.1. Precarious Work and the Healthy Work Collaborative Initiative

The term “precarious work” has been used to describe work that is “uncertain, unpredictable, and risky from the point of view of the worker” [7]. The rise in precarious work in the US can be linked to macroeconomic changes that resulted in increased global competition, which led to outsourcing of labor, weakened labor unions, and deregulation of the labor market [7]. Employers have sought to minimize costs by shifting jobs away from standard, full-time work arrangements toward a more flexible labor market. These more flexible, precarious work arrangements are characterized by low wages, a lack of protection from termination, variable work schedules, disproportionate exposure to health and safety hazards in the workplace, and working conditions that cause high psychosocial stress [2,8,9,10]. Without intervention, a growing share of workers in the US will experience precarious employment conditions, regardless of occupation [11]. 

Although studies increasingly show that these highly precarious work arrangements adversely affect the health of workers [12,13,14,15], interventions that improve the health of workers in these jobs are difficult to design and implement, given the nature of their work arrangements [16]. There is a substantial body of literature that posits that public health interventions that create the social and environmental conditions to promote and facilitate health are likely to be most effective and impactful on a population level [17,18,19]. Since many of the features of precarious work are not unique to a single occupation or to a single workplace, interventions aimed at addressing the causes of precarious work must be implemented at these broadly impactful social ecological levels. These types of interventions, typically in the form of policy, systems, and environmental (PSE) changes, are most effective when a diverse group of stakeholders are involved in intervention development and implementation, and when these stakeholders understand the problem and relevant power dynamics [17,20,21]. 

While there are several examples of successful, cross-sectoral PSE interventions to address public health issues, including tobacco control and measures to reduce automotive crashes [19], there is little evidence in the literature of similar strategies to address precarious work. Given the absence of existing best practices or evidence-based initiatives in this area, researchers at the UIC Center for Healthy Work engaged a group of multi-disciplinary stakeholders in a process designed to understand and begin to develop upstream action to address drivers of precarious work. This process, known as the Healthy Work Collaborative Initiative, involved a six-session series of instructional and planning-based activities for organizations that were interested in addressing precarious work.

The six session Healthy Work Collaborative (HWC) was part of a larger project in the UIC Center for Healthy Work. The overarching aim of this larger project was to use an action research framework to understand and address precarious work through cycles of inquiry and action planning [22]. The HWC was a component of this larger project, which was designed with an intent to increase stakeholders’ individual and organizational capacities to apply PSE strategies to address drivers of precarious work. The primary goal of the HWC was to bring together health and labor organizations to explore initiatives that may address health in the context of precarious employment. The goal of this manuscript is to report on a study that examined the role of university-based facilitation in this HWC process, conceptualized as technical assistance (TA) provided by UIC researchers. The HWC and TA in the HWC are further described below. 

UIC researchers recruited Chicago- and Illinois-based public health and healthcare organizations and their partners to participate in the six in-person HWC sessions; many participants were recruited through existing relationships between the School of Public Health researchers and representatives of these organizations. The researchers also recruited representatives of labor organizations, including Chicago-based worker centers and labor advocacy groups, to share content expertise with participants during the HWC sessions. All labor organizations represented in the HWC also had longstanding relationships with researchers in the UIC School of Public Health. All six in-person HWC sessions took place within a 10-week period in the spring and summer of 2018. 

Collaborations between university groups and outside partner organizations have been described in various contexts in the literature. Much of the existing literature on community–university partnerships focuses on opportunities for knowledge translation, or the application of research findings in the community, and service-learning and community-based research [4,6]. While the HWC model shares some of the features of community–university partnerships highlighted in the literature, such as an opportunity to co-create knowledge and develop shared research and action agendas [4], the purpose of the HWC was primarily to drive action rather than to generate knowledge.

The researchers designed the HWC using an Action Learning (AL) approach, which is an approach to problem solving that emphasizes learning through action and reflection on the results of that action [23]. AL was originally conceptualized by Reg Revans in the early 1980s, but has been adapted by others to better suit emerging learning and action needs in different contexts. One of these adaptations is that of Marquardt et al., in which AL is used with the intent to build and sustain systems-level change [24]. Similar to Revan’s original AL approach, that described by Marquardt et al. uses an iterative, participatory process, which combines scientific knowledge with evidence derived from learners’ experiences to solve complex problems [24,25]. However, unlike Revan’s approach, Marquardt et al.’s AL approach relies on AL “coaches”, or facilitators who promote critical thinking through the probing and prompting of learners throughout a process. In the HWC, UIC researchers served in this facilitator role, which is further described below. 

Activities within each HWC session were designed to build upon one another so that participants would leave with foundational knowledge and skills to begin to plan for and take action to address the drivers of precarious work. The HWC sessions were grouped into three phases (Table 1), all of which incorporated AL tools: (1) Understanding; (2) System, strategies, and approaches; and (3) Planning for action. A fourth phase, the Action phase, was not included in the HWC sessions. Each phase included two sessions. Table 1 details the purpose of each phase and the activities that were included in that phase’s sessions. 

Small stipends were provided to HWC participants to compensate for their time spent preparing for and participating in the sessions. This aligns with the community–university partnership literature that suggests that funding community engagement in university-sponsored activities both supports community involvement and demonstrates the value that the university places on community engagement [6]. Funding was also provided to representatives from local worker centers and other labor advocacy and educational organizations who served as TA providers in the HWC sessions. The various participant roles in the HWC are further described below. 

### 1.2. Participant Roles in the Healthy Work Collaborative (HWC)

Participants in the HWC sessions fell into three categories: (1) the UIC researchers who organized and facilitated the overall HWC process and served as AL facilitators; (2) representatives from labor organizing, labor advocacy organizations, and labor-focused academic organizations who attended select HWC sessions and led HWC activities during those sessions; and (3) representatives from primarily public health and healthcare organizations who attended and participated in all six HWC sessions. 

The first two groups, the UIC researchers and the representatives from labor organizations, were termed “technical assistance (TA) providers” for the HWC. Together, these TA providers engaged the largely non-labor and non-academic health-focused participants in the various HWC activities. The TA providers also engaged with individuals or small groups in other capacities within and outside of the in-person sessions as they grappled with the issue of precarious work and plans for action in their own organizational or partnership-based contexts. The role of labor expert TA is examined elsewhere (manuscript in preparation). 

While there is no empirical research pointing to an ideal structure for a TA process for moving recipients toward action, some studies point to features of TA–recipient models that make them more effective than others. Effective TA models integrate several theoretical principles, including theories of change, adult learning, consultation, and facilitation [26,27,28]. Using these principles, researchers and practitioners in several fields have conceptualized TA as a multi-tiered approach to build the capacity of individuals or organizations to achieve substantial change [29,30]. 

TA has also been classified along a continuum from less intensive, content-driven TA, to more intensive, relationship-based TA [26,29]. The intensity of TA provided to a recipient typically depends on the recipient’s needs and their desired project outcomes. Less intensive TA typically involves sharing of content or skill knowledge with the TA recipient, which is most useful when the recipient already has structures and policies in place to support PSE change [31]. This type of TA often involves fewer, less intensive TA–recipient encounters in which TA providers present information to the recipients, but do not engage in longer-term collaborative work. More intensive TA, on the other hand, requires a more sophisticated relationship between the TA provider and the recipient. In this instance, the TA provider engages in sustained, in-depth work in partnership with the TA recipient, and takes on more responsibility for the outcomes of the program that they are supporting [29]. 

In the HWC, TA provided by UIC researchers was conceptualized as more intensive, relationship-based TA, focused on facilitating behavior and systems change, while TA provided by labor experts was conceptualized as more content-driven, focused on the transfer of knowledge to participants. For the duration of the HWC, UIC TA providers divided themselves up between TA recipient groups, helping to guide TA recipients through each of the HWC activities and exercises. UIC TA providers also followed up with their TA recipient groups between HWC sessions, pointing them in the direction of resources, clarifying content from the sessions, and pushing them toward actionable next steps. This type of higher intensity TA, focused on the facilitation of learning and action planning, aligned with the role of an AL “coach” described in the AL literature [32,33]. There is some evidence that higher intensity TA, facilitation, or coaching, involving frequent check-ins and tailored supports and feedback, increases the sustained engagement of learners, or TA recipients, in later implementation or action phases [26,34]. With the HWC, UIC researchers positioned themselves in a way to both connect practitioners in different disciplines who do not already work together, and support engagement between those practitioners as they move to bring about sustainable change. Little is known about universities operating in this role, and this study aims to contribute knowledge to this gap. 

This study explores the role of TA provided by UIC researchers in the HWC process. Specifically, this study seeks to understand UIC TA providers’ perceptions of their own roles in the HWC process, facilitators, and challenges associated with these roles, and any outcomes of the HWC process that they attribute to these roles. This study also seeks to understand the perceptions of other HWC participants, including labor expert TA providers and health-focused TA recipients, regarding these same concepts. Given that TA and university–community partnerships have been identified as important mechanisms to close the “knowledge to action” gap, this study seeks to explore the importance of these factors in facilitating the learning and development of PSE change interventions in the context of the HWC. 

## 2. Materials and Methods 

UIC researchers used a mixed-methods approach to evaluate the overall HWC process. For this study, researchers used an exploratory qualitative study design with focus group and interview methodology to examine HWC participants’ perceptions of the role of TA provided by UIC in the HWC, including during the period leading up to the six sessions, the periods between sessions, and the period after the sessions. The UIC Institutional Review Board approved this study in 2018.

All 31 individuals who participated in the HWC in some capacity were invited to participate in this study. Information about the HWC participants is included in Table 2. Seven UIC TA providers participated in the HWC sessions, including two UIC faculty members, three staff members, and two student research assistants. Seven labor expert TA providers participated in at least one HWC session, and five participated in two or more sessions. Four of these labor expert TA providers represented Chicago-area worker centers, two represented national labor advocacy organizations, and one represented a labor-focused academic research center at UIC that is not part of the UIC Center for Healthy Work. A total of 17 representatives of other organizations participated in the HWC sessions as TA recipients, including representatives from local health departments (LHDs), public health and other health advocacy organizations, a hospital system, an academic institution other than UIC, a local board of health, two worker centers, and a labor union. 

Of these TA recipients, 14 came to the HWC with other partners (see the TA Recipient Groups in Table 2). These TA recipient groups focused on action planning with their partners. The remaining three TA recipients attended the HWC sessions as individual representatives of their organization, and focused their efforts on action planning within their own organization’s purview. Each TA recipient group or individual organization joined the HWC with a pre-determined focus for action planning. These foci are briefly described in Table 2. 

Several instruments were developed to obtain information about UIC TA providers’ roles in the HWC from the various HWC participants. A semi-structured focus group guide was developed to collect perspectives from the UIC facilitators immediately following the conclusion of the HWC. One semi-structured interview guide was designed to collect perspectives from the labor experts, who served as TA providers in the HWC sessions, immediately following their involvement in the HWC, and another semi-structured interview guide was designed to collect perspectives from non-labor, primarily health-focused TA recipients three months after the conclusion of the HWC sessions. Notably, both of the interview guides included questions aimed at understanding other features of the HWC and impacts of participating in the sessions. The results reported in this study focus on the aforementioned concepts around TA provided by UIC. Table 3 compares the content relevant to this study included in the interview guides and the focus group guide, all of which are further described below.

UIC TA providers were invited to participate in an in-person focus group in the days immediately following the final HWC session. The focus group guide was designed to capture UIC TA provider’s perceptions of what TA recipients gained from the HWC process, perceptions of UIC TA providers’ own roles inside and outside of the HWC sessions, and perceptions of impacts that the HWC process had on UIC TA providers’ own thinking. Labor expert TA providers were invited to participate in a follow-up phone interview in this same time frame. All TA recipients were invited to participate in a phone interview approximately three months after the final HWC session, as were TA providers who had continued to engage with TA recipients beyond the formal HWC six-meeting period. The immediate post-HWC guide and the three-month post-HWC interview guide were designed to capture labor experts’ and TA recipients’ perceptions of the same concepts as the UIC TA provider focus group guide, as well as their impressions of the HWC process more generally. TA recipients were interviewed at the three-month time point instead of immediately post-HWC to better capture ways in which the TA recipients had applied what they had learned from the HWC since the conclusion of the sessions, and any implementation of activities planned during the HWC sessions.

### Analysis

A preliminary codebook for this study was developed prior to data collection with template codes based on the study’s research questions and relevant technical assistance literature similar to the code manual development described by Fereday and Muir Cochrane [35]. Four broad code categories were included in this a priori codebook: perception of TA role, intensity of TA, impact of TA, and importance of TA. These broad categories and sub-codes within each category were included in the a priori codebook with a definition and description of each code. Emergent codes were added during the preliminary analysis steps, and are described below. The codebook used for this study was separate to that used for the overall evaluation of the HWC process. 

The in-person focus group with UIC TA providers and all phone interviews with labor expert TA providers and TA recipients were audio recorded and professionally transcribed. The transcripts were analyzed using a hybrid approach that involved both inductive and deductive coding and theme development, similar to the approach described by Fereday and Muir-Cochrane [35]. Dedoose software (Dedoose Version 7.0.23, web application for managing, analyzing, and presenting qualitative and mixed method research data, SocioCultural Research Consultants LLC, Los Angeles, CA, USA) was used for all qualitative analyses in this study. 

Immediate post-HWC interviews with labor expert TA providers and the focus group with UIC TA providers were coded by a single coder. For each transcribed interview, the following analysis protocol was used:

(1) Each full interview and the focus group transcript was read and key points were summarized in a memo. At this point, additional codes were added to the codebook based on new categories that emerged from the textual data. 

(2) Then, a priori codes and emergent codes were applied to the interview text where text segments were considered representative of and matched the definition of an individual code. 

(3) As segments of text were coded, each new excerpt was compared with segments that had previously been assigned the same code. In the event that a code did not seem to fit for both segments, a new code was added to the codebook, and relevant sections of the transcribed interview were recoded. 

(4) After all interviews were coded and additions to and refinements of the codebook were complete, a new cycle of coding began. Each interview was re-coded using the updated codebook.

(5) After the second coding cycle, final coded segments were read and subjected to a process of clustering around similar patterns. Themes were identified when all data supporting a given pattern were clustered and saturation was reached. At this stage, differences in themes across interviewees were examined.

Three-month follow-up interviews were coded by two separate coders, and a slightly different analysis protocol was used. Steps 1 and 2 from the baseline and immediate post-HWC interview protocol were followed, as described above, with both coders reading and summarizing transcribed interviews and collaboratively making additions to the codebook. Then, the following steps were completed by the two coders in lieu of steps 3–5 from the baseline and immediate post-HWC analysis protocol: After each transcribed interview was coded by both coders, coded segments were compared for agreement. In the event that the two coders did not agree on coding for a particular segment, they discussed the segment and attempted to come to agreement as to which code(s) should be applied. In the event that the two coders could not come to agreement, a third coder was asked to code the interview and discuss applied codes with the original two coders. Additionally, in the event that no codes seemed to fit a given segment, a new code was added to the codebook, and relevant sections of the transcribed interview were recoded. Both coders reviewed already coded interviews for a comparison of applied codes and recoded those interviews as needed to reflect codebook updates. Final coded segments were read and a process of clustering around similar patterns and themes began. At this stage, differences in themes across participant type were examined.

## 3. Results

A total of 22 HWC participants (71%) participated in either the in-person focus group or at least one follow-up phone interview after the conclusion of the HWC. The immediate post-HWC focus group lasted approximately 90 minutes and was conducted in person, while the immediate post-HWC and three-month post-HWC follow-up interviews lasted approximately 60 min and were conducted by phone. 

Seven UIC TA providers participated in the immediate post-HWC focus group, representing all but one of the UIC representatives who helped to facilitate the HWC process. One UIC representative (the first author on this paper) facilitated but did not participate in the focus group. Five labor expert TA providers participated in interviews immediately post-HWC, and two of these TA providers also participated in three-month post-HWC follow-up interviews. The two TA providers who participated in three-month follow-up interviews had substantial, continued involvement with at least one other non-TA HWC participant beyond the six HWC sessions, either in the form of more tailored and intensive TA provision or in the form a formalized partnership. Ten non-labor, primarily health-focused TA recipients also participated in three-month post-HWC follow-up interviews.

UIC TA providers, labor expert TA providers, and TA recipients shared a variety of perceptions of UIC TA in the HWC. Findings from the focus group and interviews are organized under the following broad categories: UIC’s role in the HWC, facilitators and challenges associated with UIC’s role, impacts of UIC’s provision of TA, and future roles for UIC beyond the HWC. 

### 3.1. Role of UIC TA in the HWC

All participants, including UIC TA providers, labor expert TA providers, and TA recipients reflected on the utility of UIC researchers as TA providers in the HWC. Three main themes emerged from the focus group and interview data: (1) the value of UIC’s role as a convener of the HWC; (2) UIC TA providers’ ability to facilitate learning by guiding TA recipients through the HWC activities and holding them accountable to the next steps; and (3) UIC researchers’ ability to both fill a gap in the literature and aid in the development of actions to address a complex issue. Each of these themes is further described below.

#### 3.1.1. UIC’s Role in Convening the HWC

In individual interviews, TA recipients and labor expert TA providers shared their perspectives of UIC’s role as a convener of the HWC. Generally, interviewees noted that UIC was an appropriate connector and host for such a process, given the value that UIC as an institution places on community engagement. Interviewees described their own experiences interacting with faculty and staff at the university, and several highlighted explicit value statements put forth by university groups that reinforce UIC’s commitment to community-engaged activities. One TA recipient mentioned the UIC School of Public Health, which houses the Center for Healthy Work, as being particularly committed to community engagement:

“So I think that one of the public health school’s missions, or part of the mission, is to be engaged with the community. And I think that this is one very strong way of doing it.”—TA recipient.

Interviewees also highlighted the rigor that a university can bring to an initiative such as the HWC. Several interviewees noted the reputation of the UIC School of Public Health and its recognition as a leading research institution in Chicago. At least one interviewee described the value of having a public health perspective when planning for action around upstream issues such as work:

“I think there is certain rigor to having it, in a public health perspective, that maybe in a limited way could have come from some of the other participants in the collaborative… [UIC TA providers] brought that.”—TA recipient.

#### 3.1.2. UIC’s Role in Facilitating Learning

An important observation of UIC TA providers’ own role in the HWC was that of facilitating a shared language and fostering opportunities for open dialogue about the issues related to precarious work for all participants. UIC TA providers generally agreed that establishing a definition of precarious work early on in the HWC sessions helped to facilitate engagement in subsequent HWC activities and deeper dialogue between the various participants. One UIC TA provider described both UIC and labor expert TA providers’ role in establishing this shared language:

“So I think it was a skill that people were able to find a shared language, and I think we helped facilitate that along with the TA providers, to be able to talk to one another.”—UIC TA provider.

UIC TA providers further described their role as “pushing” or “coaching” TA recipients toward action as they progressed through the HWC sessions. Several UIC TA providers shared examples of ways in which they had helped TA recipients develop action steps based on what they had learned or created in HWC session activities; for example, one UIC TA provider had helped their group build a small action plan based on the Theory of Change that the group had developed during one of the HWC sessions. Several UIC TA providers noted that TA recipients were seemingly appreciative of this type of TA-led facilitation and encouragement, as summarized by one UIC TA provider below:

“I did hear quite a bit that having somebody to push them to help them focus, give them that extra support… They wouldn’t be doing it, without that push. They need the push. They need the… And, I don’t mean pushing them out the door. But, th… to help encourage, to build their self-advocacy/capacity.”—UIC TA provider.

In interviews, many TA recipients shared similar reflections of the utility of UIC TA providers’ facilitation or “pushing” of TA recipients throughout the HWC process. Several TA recipients described specific interactions with UIC TA providers during or between HWC sessions in which the TA provider had helped them to further refine or develop tools or plans to move the recipients toward action. One TA recipient described their experience as follows:

“I liked the idea that you had a staff person that was sort of assigned to each group, because it really kept us together, and then you organized us. You made sure we had meetings, and we decided to have a little pre-meeting before the actual training sessions, and you really facilitated sort of all of the logistics, as well as providing leadership in the groups. And I think we loved working with the folks that we were working with.”—TA recipient.

TA recipients also recognized UIC TA providers’ roles in guiding them toward a more profound understanding of precarious work and TA recipients’ own roles in addressing its drivers. Several TA recipients noted the ways in which UIC TA providers helped TA recipients to think about the issues without being overly prescriptive or forceful in what their takeaways should be. One TA recipient described their experiences with UIC TA providers as follows:

“One of the things I liked is that with [the] UIC facilitator and UIC facilitator and everybody, you all guided. You don’t imprint on it… And it’s a great way to learn. And you helped guide people to where, I think where we should have gotten to without saying, you know, you let us have a learning experience, that’s what I guess I’m trying to say, without handing us a syllabus and saying, ‘You’re going to be at this point, this point,’ you know what I’m saying? And so I really liked that approach. And it’s really very beneficial.”—TA recipient.

#### 3.1.3. UIC’s Role in Contributing Evidence and Facilitating Action

In the focus group, UIC TA providers reflected on the factors that made their role in organizing and coordinating the HWC feasible and appropriate. Several UIC TA providers described the gap in the literature around PSE strategies to address the drivers of precarious work, and how this gap presented an opportunity for the researchers in the UIC Center for Healthy Work to gather contributing evidence in this arena via the HWC. One UIC TA provider summarized these sentiments below:

“…it’s about building the evidence that doesn’t exist, there is not good evidence around how to do PSE change around in particular precarious work for sustainable change, and that’s what we’ve been trying to do and we are documenting it, we’re building evidence, we’re adapting theory based on feedback for practice and integrating it to do something we hope is impactful.”—UIC TA provider.

UIC TA providers also engaged in a discussion around academic expectations and needs for evidence building that allow for the dedicated time and funding to support an initiative such as the HWC. At least one UIC TA provider mentioned the need to respond to the expectations of the funding agency for the Center for Healthy Work by collecting data and producing products for dissemination, which is made possible through the engagement of other stakeholders in the HWC.

“…we have to keep the funders in mind and research and building evidence in mind… So having a product, something that can be disseminated widely or policy change, environmental change, having something happen that can be counted, that’s [the funder’s] perspective.”—UIC TA provider.

### 3.2. Facilitators and Challenges Associated with UIC TA Role

Several themes emerged from the data regarding facilitators and challenges associated with the TA role that UIC researchers played in the HWC. Existing relationships between UIC researchers and representatives from the organization that participated in the HWC, and UIC’s knowledge of the participants’ needs and related opportunities emerged as facilitators associated with the UIC researchers’ role; in contrast, constraints related to time, content, planning, and limitations to TA control emerged as challenges associated with this role. These facilitators and challenges are described below. 

#### 3.2.1. Existing Relationships

Labor expert TA providers and TA recipients described their existing relationships with UIC researchers as a catalyst to their involvement in the HWC. All labor expert TA providers described longstanding relationships with UIC faculty and staff in the Environmental and Occupational Health Division of the School of Public Health, while most of the non-labor TA recipients described existing relationships with faculty and staff at the MidAmerica Center for Public Health Practice, also in the School of Public Health, which provided training to public health professionals. Representatives from both School of Public Health groups were involved in the planning and coordinating of the HWC. One TA recipient described their relationship with UIC and their decision to participate in the HWC:

“We’re fortunate enough to have a long-standing working relationship with UIC… So I heard about the collaborative from [UIC facilitators], and we talked about some of the work that was going to be done and what the overall, I guess what the health outcomes might be. There was [sic] some issues that I’ve kind of wanted to work on, and so I just said, yeah, I kind of was interested in pursuing this.”—TA recipient.

Several interviewees indicated that they felt UIC researchers had their organization’s best interests and needs in mind when soliciting their involvement in something such as the HWC. A labor expert TA provider summarized this sentiment:

“…we have relationships with individuals and the departments that span all my time here… we already have an idea of what kind of things that it would involve, or yeah, there’s less uncertainty about, “Well, would this be a good use of my time?” That sort of thing ‘cause we already have the relationship and are accustomed to working together. I think part of it is just experience is a factor in our decision making here to engage, being that we already have experience together.”—Labor expert TA provider.

#### 3.2.2. UIC’s Capacity to Recognize Needs and Opportunities

Additionally, several interviewees described the university’s unique capacity to recognize the need for and engage a diverse group of stakeholders to collaboratively learn about and plan for upstream action to address drivers of precarious work. Several interviewees described the unique features of the university as a convener, including its commitment to community engagement and its existing relationships with community organizations (further described below) that made the HWC especially impactful in a way that it would not have been without UIC’s involvement. One interviewee summarized these sentiments below:

“I doubt we would have had the same kind of people, diversity of entities in the room and in conversation. Without you all… I doubt the conversation would have happened without the (HWC), the grant funding which all happened behind it.”—TA recipient.

At least one TA recipient also noted that the representatives in the HWC would not have had the opportunity to connect if it were not for the convening of the HWC.

“It is really helpful to bring groups together who haven’t worked together before and who we may not always think of—and see how it ties back to our work—unless we get connected and seek it out on our own, which we don’t really have time for, we don’t have access to these new relationships.”—Labor expert TA provider.

#### 3.2.3. Time and Content Balance

Despite the many touted benefits of UIC’s TA provision in the HWC, participants described some of the limitations, challenges, and opportunities for change given their experience in this HWC process. Many of the TA recipients, in particular, described the challenges of digesting so much new content in such a short period of time. Some TA recipients also felt that there was not enough time built in to reflect upon and apply what they had learned in the HWC sessions. One TA recipient summarized these sentiments below:

“It was super structured and a lot of stuff over a short timeline. There was a balance that it needed to be structured so it didn’t lose the thread… But it still was quite a bit.”—TA recipient.

Several UIC TA providers also attributed challenges in timing with what TA recipients were able to accomplish through the HWC process. UIC TA providers felt that the readiness of TA recipients to both engage with a complex new issue such as precarious work and actively plan implementable interventions outside of the HWC sessions varied between groups and individual TA recipients. One UIC TA provider noted that the timing of the HWC was based solely on UIC researchers’ own needs and not on the needs of participants, including both the readiness piece and the amount of time participants, including TA recipients and labor expert TA providers, needed in between sessions to digest information and prepare for subsequent sessions. One UIC TA provider summarized these observations below:

“I think the timing issue was really a very important factor in influencing what went on and what went well for some and what didn’t go well for others.”—UIC TA provider.

#### 3.2.4. Time and Planning Constraints

UIC TA providers noted some of the challenges related to the tight HWC timeline, both in terms of what content could be covered in the sessions and in terms of limitations in time to plan the sessions. Several UIC TA providers shared the challenges that stemmed from working within a time-bound grant structure, in which funds allocated to activities such as the HWC needed to be spent down within a short time frame. This presented problems with HWC planning, limiting UIC TA providers’ abilities to engage labor expert TA providers in much of the planning in advance of the meetings themselves:

“…ultimately we only had so much time to devote to developing this curriculum and structuring these meetings that there are certain things with the curriculum that I think we may have done differently that would facilitate learning in a different way. I wonder if we had brought in all of the… if they had the capacity to do this, if we had brought in all of the TA providers from the get go to develop a curriculum in a more collaborative way.”—UIC TA provider.

#### 3.2.5. Limitations of UIC TA Role

Beyond the challenges related to content load and timing, UIC TA providers shared several limitations of what they as TA providers were able to bring about through the HWC process. Although they were able to provide tools to TA recipients, follow up with them between sessions, and push them to focus on particularly relevant content or action steps, UIC TA providers could not force TA recipients to actually move toward action. Many UIC TA providers described an obvious shift in TA recipients’ thinking over the course of the HWC sessions, but in many cases felt that it was not apparent how those TA recipients will actually move toward action post-process. One UIC TA provider described this challenge below:

“The “doing” part in their case, I struggled with … so I don’t know what else I could have done or you could have done. We literally handed them a lot of stuff and I couldn’t get them to really put a plan together ultimately, in terms of what was next.”—UIC TA provider.

Another limitation encountered by UIC TA providers was their ability to promote diverse partnerships between TA recipients in the HWC. While some TA recipients entered the HWC with pre-determined partners (e.g., representatives of other organizations interested in developing interventions to address issues of shared interest), others began and ended the HWC process as individual representatives of their own organizations. One UIC TA provider, who worked primarily with one such individual, described this as an observed limitation during the HWC process:

“I think [what] the other groups show was that coming in with a team with a diversity of voices really does make a difference, that that can foster learning… I think he had some shift in his ideas but I don’t it was as much as with the groups where people came in as a diverse team.”—UIC TA provider.

### 3.3. Impacts of UIC TA Provision

Despite some limitations to UIC TA providers’ roles in the HWC process, UIC TA providers, labor expert TA providers, and TA recipients were able to articulate a number of impacts attributed to TA provision by UIC researchers in the HWC. Two themes that emerged from the data included pushing TA recipients toward a more concrete understanding of precarious work and holding TA recipients accountable to next steps throughout the HWC process. Both themes are described below. 

#### 3.3.1. Shifts from Abstract to Concrete Understanding of Precarious Work

In the focus group, UIC TA providers described the various ways in which they helped guide TA recipients toward a deeper understanding of the drivers and manifestations of precarious work, both within the HWC sessions and in follow-up calls with TA recipients between sessions. One UIC TA provider observed the most significant changes in TA recipients’ understanding of the issues when debriefing the previous week’s session by phone:

“I think for some of the groups that I was working with, it was a big transition between abstraction and like recognizing, “Yeah, this is an issue,” and then putting pen to paper and actually devising a plan and having concrete ways to talk about it and to address it. I think that those changes have been the most in our individual phone calls with them.”—UIC TA provider.

#### 3.3.2. Accountability and Resultant Shifts toward Action 

In addition to encouraging or “pushing” TA recipients toward actionable next steps, UIC TA providers and TA recipients agreed that UIC TA also helped hold individuals and groups accountable to those next steps. Several UIC TA providers described the utility in scheduling follow-up conversations, typically by phone, in between HWC sessions as means to check in with TA recipients about agreed upon next steps. One UIC TA provider highlighted this accountability role as particularly impactful for a TA recipient they were working with:

“I think having someone to hold him accountable and I feel like the same as the case with the other team too, being held accountable for something made a big difference.”—UIC TA provider.

### 3.4. Role of UIC beyond HWC

Focus group and interview data revealed a range of anticipated needs for UIC TA provision post-HWC. In the focus group, which took place several days after the conclusion of the HWC, several UIC TA providers speculated that going forward, many of the TA recipients would need additional supports as they continued to both digest information from the HWC and move forward in their plans for action. At least one UIC TA provider expressed a feeling that TA recipients would require little content-related TA beyond the HWC sessions, but would instead require substantial guidance and continued structure and encouragement from UIC TA providers:

“I think going forward, it seems to me as though there may have to be less of a desire for the other TA providers and more of a desire for things that we can do. Which is sort of helping them navigate things and connect them to other resources that may not be one of our TA providers…”—UIC TA provider.

TA recipients echoed these sentiments in their interviews, indicating that they would value and benefit from additional facilitation and related supports from UIC TA providers moving forward. Some TA recipients went so far as to describe a specific role for UIC in the implementation of their planned actions, with some describing UIC TA providers serving in coordination and evaluative capacity. One TA recipient commented on the utility of having UIC TA support beyond the HWC:

“I think just having [UIC] as technical assistance providers as we move our project forward… it would be really helpful if our team doesn’t have to develop the next phases on our own or come up with the ideas… I think our group is open enough to your feedback on the direction to take the project.”—TA recipient.

## 4. Discussion

This study examined the perceptions of HWC participants, including UIC TA providers, labor expert TA providers, and TA recipients of UIC researchers’ TA role throughout the HWC process. The findings centered on HWC participants’ perceptions of the appropriateness and utility of UIC’s role as TA in the HWC, challenges encountered in this TA provider–recipient model, and potential next steps for UIC’s involvement with TA recipients beyond the HWC sessions. The findings provide insight into the role of a university, such as UIC, in convening a learning and action planning initiative such as the HWC, and highlight the impacts of UIC TA providers’ engagement with other participants throughout the HWC process. UIC’s experience in convening and facilitating the HWC sheds light on factors that contributed to participants’ perceptions of the success of the university-facilitated TA provider–recipient model for learning and action development, which may be useful to other universities or similarly positioned organizations interested in engaging diverse stakeholders with the aim of facilitating PSE change. Data from this study suggest that this unique model helped to prepare representatives of various organizations to develop PSE change initiatives to address the complexities of precarious work. 

This study provides important insight into how universities, such as UIC, can position themselves to support non-academic organizations across sectors and levels to facilitate evidence-informed development and the implementation of actions to address complex problems such as precarious work. The data from this study highlight the utility of having a community-engaged university bring together organizations that have existing relationships with the university, but do not necessarily have existing relationships with one another. The data also highlight the benefits and challenges of having university researchers play a TA role in a process such as the HWC, and suggest ways in which university researchers might be involved beyond initial capacity building activities to support the implementation of PSE change. 

Findings that highlight the value that HWC participants placed on UIC’s role as a research-focused and community-engaged institution offer support to UIC’s decision to organize the HWC and convene its various participants in six in-person sessions. These findings align with much of the community–university partnership literature, which details community engagement with university researchers as a means for knowledge translation and the development of shared action agendas [4]. This supports UIC’s role in putting together an initiative that has the potential to help close a knowledge-to-action gap, although this initiative differs from many of the examples in the literature. Unlike other community–university partnerships, the HWC relied on the expertise of outside TA providers, in this case labor experts, to share knowledge with the groups who are well positioned to implement interventions to address a complex problem, in this case the multi-faceted drivers of precarious work. UIC researchers’ roles as facilitators differs from the more traditional knowledge-sharing role described in much of this literature. 

Due to longstanding relationships with individuals involved in occupational health research and public health practice groups at UIC, labor expert TA providers and TA recipients described a level of trust and reciprocity that were vital to their decisions to participate in the HWC. These findings indicate that UIC was uniquely positioned to convene and facilitate the HWC, suggesting that the HWC participants may not have otherwise willingly participated in such an initiative. It is unlikely that without the HWC, participants would have interacted with one another at all, further highlighting the importance of UIC’s role in supporting important steps toward PSE change. Without strong, pre-existing relationships between UIC researchers and members of the various organizations that were represented in the HWC, these representatives may not have decided to commit the time and resources to participate the inaugural HWC initiative. The time and effort that UIC researchers put into developing and maintaining their relationships with the labor- and health-focused organizations that ultimately agreed to participate in the HWC, either as TA providers or TA recipients, cannot be overlooked as an important step in facilitating diverse engagement and commitment to participate in a pilot initiative such as the HWC. 

In addition to UIC’s role as a convener of the HWC, data from the focus group and interviews reveal several perceptions of the function of UIC TA in the HWC sessions. These functions, from providing guidance, facilitation, encouragement, and accountability to TA recipients, display the range of intensity of TA provided by UIC researchers in the HWC model. This intensity differed from that described in the TA literature, with UIC TA providers serving in a capacity that might be likened to that of a coach or accountability manager instead of a role in which the TA provider takes on responsibility for some of the work. The HWC model seemed nevertheless effective for TA recipients, many of whom attributed their progress in digesting HWC content and planning for next steps of the involvement of UIC TA providers. This suggests that TA as it is described in the literature does not fit the HWC’s model, and perhaps an expanded definition of TA is needed. Further, this suggests that university facilitation using AL, in a model such as the HWC, may be effective in increasing knowledge to action. This aligns with calls for capacity-building initiatives to foster more effective public health practice to address complex issues such as precarious work [36]. 

These findings did highlight some of the limitations of this UIC TA model. Many of the limitations described by participants revolved around the the timing and tight timeline of the HWC, both of which resulted from constraints of operating within a time-bound grant period. UIC TA providers described the challenges of planning and developing the HWC curriculum in such a short time period, which limited opportunities for cooperative planning between UIC and labor expert TA providers. Likewise, labor expert TA providers noted the challenges of not being involved in the planning of each session. This particular issue highlights the limitations of having a university group, which relies on grant funding, designing, and hosting such an initiative, given many of the factors, such as timing and funding, are determined by the funder and are out of their immediate control. 

Finally, findings from the focus group and interviews suggested that TA recipients would value and benefit from UIC TA beyond the HWC sessions. This finding highlights some of the ways in which TA recipient organizations were underprepared for action following the HWC, likely requiring additional guidance and supports to move their plans forward. This finding also highlights the importance of sustained engagement between university groups, such as the UIC TA providers in the HWC, and community groups, which is mirrored in the community–university partnership literature [4]. 

While this manuscript describes the role of university-provided TA in developing strategies for addressing precarious work, evaluation of the HWC in promoting sustainable relationships and partnerships is ongoing. The impact of the HWC on organizational priorities and on process and systems change to better address precarious employment is also an area of ongoing and future research.

### Limitations

There are a number of limitations in this study, including low participation in study components and the involvement of the author in the HWC process. Since the HWC process was a pilot, there was only a small number of representatives in both TA provider and TA recipient roles who participated in this study, and approximately 30% did not participate in interviews. Since the TA provider sample was especially small (seven UIC TA providers and seven labor expert TA providers), attempts were made to accommodate varying schedules and allow for participation in interviews or the focus group at times that best suited the TA providers. For TA recipients, similar efforts were made to ensure that at least one representative from each team (see Table 1) was interviewed to capture the team’s experiences. An additional limitation was there were no opportunities to compare findings from this study to another similar collaborative process with multiple TA providers and TA recipients, as similar examples of TA provider–recipient models were not found in the literature. 

Another potential limitation of this study is the author’s involvement in the HWC as one of the UIC TA providers who helped with the design and facilitation of the HWC process. This presents a potential bias, both due to the author’s own involvement and perceptions of the HWC and the potential bias in interviewees’ responses to interview questions, given their knowledge of my role in the HWC process. To partially address this limitation, assurances were made to participants that their data would be both de-identified and reported in the aggregate, and would not be shared outside of the UIC research team. Further, another UIC TA provider conducted interviews with the TA recipients who the author interacted with most directly in the HWC sessions. Although the author did facilitate the focus group with other UIC TA providers, she did not participate in the focus group herself (i.e., she did not share her own perceptions of the HWC process and the role of TA). The author worked with an external colleague to code and debrief transcribed data to both reflect upon and document potential biases and subjectivities. 

## 5. Conclusions

The complex problems that workers face, especially those in precarious work arrangements, demand innovative and comprehensive solutions. The *Total Worker Health^®^* model recognizes the need for research and practice to improve the health of workers, and TWH Centers for Excellence, such as the Center for Healthy Work at UIC, are tasked with understanding the conditions that workers face and developing strategies to improve those conditions through multi-disciplinary projects. The findings from this study, which focuses on an initiative at the UIC Center for Healthy Work, highlight the utility of university facilitation in engaging diverse stakeholders in learning and action planning, in the context of a process rooted in Action Learning, to promote action to address drivers of precarious work. 

## Figures and Tables

**Table 1:** Healthy Work Collaborative (HWC) Initiative.

Phase	Purpose of Cycle in the HWC	Aligning HWC Activities
Understanding	Gather information and begin to develop a shared understanding of precarious work.	Presentations and Q&A with panel of experts * Root cause analysis and creation of a rich picture diagram (systems map).
System, strategies, and approaches	Analyze and interpret data from the “Understanding” phase and further develop a shared understanding of precarious work and approaches to address it.	Framing and stakeholder exercises. Power analysis and mapping exercise.
Planning for action	Begin to develop a plan for action to address drivers of precarious work based on the shared understanding of precarious work from the previous cycles.	Past, current, and future state exercise. Development of a Theory of Change.
Action	Implement the plan for action developed during the previous phase. The “Action” phase was not part of the HWC sessions.	The “Action” phase was not part of the HWC sessions, but data collection for this study occurred during this phase.

* Experts included representatives from local worker centers and other labor advocacy organizations, as well as labor-focused academic partners from outside of the University of Illinois at Chicago (UIC) Center for Healthy Work. These experts are further described under “Technical Assistance (TA) in the Healthy Work Collaborative (HWC)”.

**Table 2:** HWC Participants. TA: technical assistance, UIC: University of Illinois at Chicago.

**TA Provider**	**Individuals/Organizations Represented (*N* Individual Representatives)**	**Focus of TA Provision**
UIC TA Providers	Faculty (2) Staff (3) Students (2)	Process TA; organized HWC process and engaged TA recipients directly in in-depth discussions and action-planning activities using an AL approach. Clarified content and pushed TA recipients to move toward action.
Labor Expert TA Providers	Worker Centers (4) * Advocacy Orgs (2) Academic Orgs (1)	Content-focused TA; focused on the transfer of knowledge to TA recipients. Engaged TA recipients in presentations and discussions about precarious work and skills and strategies to address it.
**TA Recipient Groups**	**Individuals/Organizations Represented (N individual representatives)**	**Focus for Action Planning**
Rural	Local Health Department (LHD) (2), workforce development org (1), government representative (1)	Develop interventions to support health and well-being of precarious workers in rural county.
Hospital–Legal–Labor	Hospital system (1), legal organization (1), worker center (1) *	Identify precarious workers who enter hospital system and connect with appropriate legal and other support services.
Public Health Advocacy–Academic	Public health advocacy organization (1), academic institution (1)	Improve community health worker employment structures across the state of Illinois.
LHD-Labor 1	LHD (1), worker center (1)	Develop strategies to enforce minimum wage and sick-leave ordinances at county level.
LHD-Labor 2	LHD (1), worker center (1) *	Develop strategies for LHD’s enforcement of labor standards during routine restaurant inspections.
**TA Recipient Individuals**	**Individuals/Organizations Represented (N individual representatives)**	**Focus for Action Planning**
Health Advocacy 1	Health advocacy organization (1)	Develop paid internship model focused on equity and inclusion.
Health Advocacy 2	Health advocacy organization (1)	Explore strategies to include precarious workers in workplace wellness programs.
Labor Union	Labor union (1)	Develop strategies to organize low-wage healthcare workers.

* Note: Two of the worker center representatives served in both labor expert presenter roles and participant team roles in the HWC initiative.

**Table 3:** Data Collection Instruments.

Instrument	Intended Audience	Key Constructs for this Study
Immediate Post-HWC Focus Group Guide	UIC TA providers (process facilitators).	Observed impacts of UIC TA provider engagement with other participants. Perceptions of value of UIC TA provider role. Challenges and facilitators to HWC TA providers–recipient model. Opportunities for engagement beyond HWC.
Immediate Post-HWC Interview Guide	Labor expert TA providers (content experts).	Experiences with UIC TA providers; observed and experienced impacts of all participants’ engagement with UIC TA providers. Perceptions of value of UIC TA provider role. Challenges and facilitators to HWC TA providers–recipient model. Opportunities for engagement beyond HWC.
Three-Month Post-HWC Interview Guide	All non-TA provider participants (TA recipients). Labor expert TA providers involved with TA recipients beyond HWC sessions.	Experiences with UIC TA providers; impacts of engagement with UIC TA providers. Perceptions of value of UIC TA provider role. Challenges and facilitators to HWC TA providers–recipient model. Opportunities for engagement beyond HWC.
